# A case of biliary tract infection with *Streptococcus cristatus* bacteremia

**DOI:** 10.1016/j.idcr.2026.e02586

**Published:** 2026-04-22

**Authors:** Shohei Katsuno, Youta Takano, Ryosuke Nakamori

**Affiliations:** a Department of Pharmacy, Nagano Chuo Hospital, Nagano City, Japan; b Department of Clinical Laboratory, Nagano Chuo Hospital, Nagano, Japan; c Department of Gastroenterology, Nagano Chuo Hospital, 1570 Nishitsuruga-machi, Nagano, Nagano, Japan

**Keywords:** *Streptococcus cristatus*, Biliary tract infection, Bacteremia

## Abstract

**Background:**

*Streptococcus cristatus* is an oral commensal organism belonging to the viridans group *streptococci*. Although generally nonpathogenic, recent reports have highlighted its potential to cause invasive infections. However, gastrointestinal infections due to this organism have not been previously documented. We report the first case of a biliary tract infection with *S. cristatus* bacteremia, highlighting its clinical relevance.

**Case presentation:**

A 74-year-old Japanese man with hypertension and diabetes mellitus presented with severe epigastric pain and vomiting. Contrast-enhanced computed tomography confirmed choledocholithiasis, revealing common bile duct dilatation with an 8-mm stone near the ampulla of Vater. Blood cultures obtained on admission grew gram-positive cocci in chains, later identified as *S. cristatus* by 16S rRNA gene sequencing. The isolate was susceptible to beta-lactam antibiotics. Intravenous cefmetazole was administered for 6 days. Endoscopic retrograde cholangiopancreatography with sphincterotomy was performed on hospital days 2 and 5, achieving biliary drainage and stone removal. The patient recovered uneventfully and was discharged without sequelae on day 10.

**Conclusions:**

This case represents the first documented biliary tract infection associated with *S. cristatus* bacteremia. Accurate species-level identification is critical, as it helps recognize the pathogenic potential of organisms traditionally considered commensal.

## Background

*Streptococcus cristatus* is a gram-positive, catalase-negative, chain-forming coccus [1]. It is typically found as a commensal organism in the oral cavity and throat [1]. Recent research has demonstrated that *S. cristatus* can modulate oral microbial community virulence by inhibiting *Porphyromonas gingivalis* [2].

Despite its commensal status, recent case reports have highlighted the potential of *S. cristatus* to cause invasive infections. Reported cases include neonatal septic arthritis [1], infective endocarditis [3], [4], vertebral osteomyelitis [5], bacteremia [6], and spondylodiscitis [7].

The pathogenesis of invasive *S. cristatus* infection appears to involve translocation from the oral niche following mucosal disruption or in patients with predisposing factors [6]. However, the specific virulence determinants responsible for invasive disease in humans remain poorly characterized [4]. Here, we present the first case of biliary tract infection with *S. cristatus* bacteremia.

## Case presentation

A 74-year-old Japanese man with a 2-week history of epigastric pain was admitted to our hospital. He had been receiving treatment for hypertension and diabetes mellitus and had no history of biliary tract disease or recent dental procedures.

Two weeks prior to hospitalization, he developed epigastric pain accompanied by vomiting. The symptoms improved for approximately 1 week with histamine-2 receptor antagonists. However, 2 days before admission, the epigastric pain with vomiting recurred in the evening. On the day of admission, he presented with severe epigastric pain and vomiting.

On admission, his vital signs showed hypertension (blood pressure 163/78 mmHg), tachycardia (heart rate 91 beats per minute), and a body temperature of 36.5°C (likely reduced by prehospital antipyretic and analgesic administration). Cardiovascular examination revealed no murmurs, and pulmonary examination was unremarkable. Abdominal examination showed a flat, soft abdomen with tenderness in the epigastric region. No muscular guarding or jaundice was observed. Bowel sounds were diminished.

Laboratory studies revealed leukocytosis, with a white blood cell count of 11,060/μL (normal: 3,300–8,600/μL). C-reactive protein was mildly elevated at 0.48 mg/dL (normal: <0.14 mg/dL). Liver function tests showed elevated total bilirubin at 2.6 mg/dL (normal: 0.4–1.5 mg/dL) and direct bilirubin at 1.9 mg/dL (normal: <0.4 mg/dL), indicating conjugated hyperbilirubinemia. Hepatic transaminases were elevated, with aspartate aminotransferase at 165 U/L (normal: 13–30 U/L) and alanine aminotransferase at 143 U/L (normal: 10–42 U/L). Gamma-glutamyl transferase was markedly elevated at 653 U/L (normal: 13–64 U/L), indicative of biliary obstruction.

Contrast-enhanced computed tomography (CT) of the abdomen revealed dilatation of the common bile duct, intrahepatic bile ducts, and pancreatic duct. An 8-mm stone was identified near the ampulla of Vater, confirming choledocholithiasis causing biliary obstruction. No abscess formation was observed on CT.

**Fig. 1:**
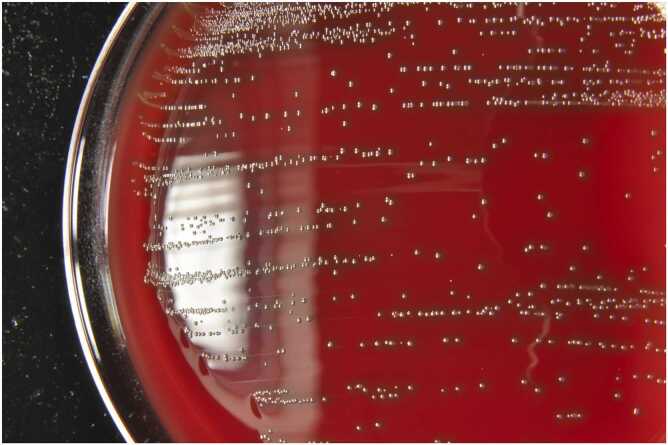
The colony of *S. cristatus* was cultivated on 5% sheep blood agar following incubation at 35°C for a duration of 18 h within a 5% CO_2_ atmosphere.

Blood culture (two sets) obtained on admission grew gram-positive cocci in chains. It was determined that all four blood culture bottles were positive in the initial 13–18-hour period of incubation. The isolate exhibited α-haemolysis on 5% sheep blood agar after incubation at 35°C for 18 h in 5% CO_2_ (Fig. 1). It was initially identified as *Streptococcus acidominimus* using the BD Phoenix™ 50 system. However, as this species is difficult to identify accurately using this instrument, genetic testing was performed. The organism was identified as *S. cristatus* by 16S ribosomal RNA (rRNA) gene sequencing followed by Basic Local Alignment Search Tool analysis, showing 99.7% homology with American Type Culture Collection 51100, the type strain of *S. cristatus* [8].

Antimicrobial susceptibility testing was performed using the BD Phoenix™ Streptococci SMICIID-8 panel, and the results were interpreted according to the Clinical and Laboratory Standards Institute (CLSI) M100-S28 guidelines (including breakpoints for viridans group streptococci [VGS], non-meningitis isolates). Drug susceptibility testing revealed minimum inhibitory concentrations (MICs) as follows: penicillin G ≤ 0.06 μg/mL; ampicillin ≤ 0.25 μg/mL; ceftriaxone ≤ 0.25 μg/mL; meropenem ≤ 0.06 μg/mL; clindamycin ≤ 0.03 μg/mL; and levofloxacin ≤ 0.5 μg/mL. The isolate was susceptible to most of the tested antibiotics.

**Fig. 2:**
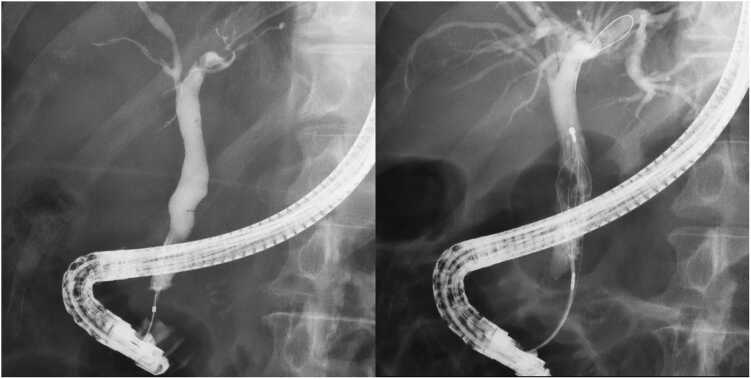
The second ERCP images before and after for bile duct stone removal.

Intravenous cefmetazole (CMZ) (1 g twice daily) was administered for 6 days. Endoscopic retrograde cholangiopancreatography (ERCP) with endoscopic sphincterotomy (EST) was performed on hospital days 2 and 5 (Fig. 2). Bile culture was not performed, as the clinical team prioritized immediate biliary drainage and stone removal to achieve source control. Because the patient received acetaminophen intermittently after admission for pain control and prevention of fever recurrence, the body temperature did not exceed 37.6°C during hospitalization. The patient was discharged on day 10 without sequelae, and no further follow-up was conducted.

## Discussion and conclusions

A PubMed search conducted in December 2025 using the terms “*S. cristatus*” and biliary tract infections (e.g., cholangitis, cholecystitis) identified no relevant case reports. When the search was expanded to include the entire VGS, rare cases of liver abscess caused by the *Streptococcus anginosus* group (SAG) were identified [9]. Before the widespread use of genetic testing, misidentification could not be entirely excluded. However, SAG is well known for its propensity to cause abscesses, and the previously reported cases were consistent with this characteristic. The present case represents the first documented instance of biliary tract infection due to this microorganism. Several other invasive infections associated with *S. cristatus* have been reported.

Native valve infective endocarditis was reported in a 68-year-old man who developed multiple aortic valve vegetations and splenic embolization; he was successfully treated with benzylpenicillin and gentamicin, followed by valve replacement surgery [3]. Another case involved a 72-year-old man who developed *S. cristatus* bacteremia and presumed endocarditis after aortic valve replacement; he received 6 weeks of ceftriaxone. Although he initially improved, he later developed progressive aortic insufficiency, and prosthetic valve endocarditis caused by *S. cristatus* was confirmed by 16S rRNA sequencing 8 months later, necessitating repeat valve replacement surgery [4].

Vertebral osteomyelitis with discitis was reported in a 66-year-old man with chronic back pain who required prolonged antibiotic therapy for spinal infection due to *S. cristatus* [5]*.* In another report, a 59-year-old man with end-stage cryptogenic cirrhosis and poor oral hygiene developed *S. cristatus* bacteremia. He was treated with 8 days of intravenous ceftriaxone (1 g/day) followed by six days of oral cefpodoxime (400 mg/day). Echocardiography showed new mild-to-moderate aortic regurgitation, fulfilling Modified Duke Criteria for “possible endocarditis”; however, clinicians elected not to pursue endocarditis-specific treatment [6].

A case of spondylodiscitis due to *S. cristatus* was also identified by 16S rRNA sequencing, requiring prolonged antibiotic therapy appropriate for bone and joint infection [7]. Furthermore, neonatal septic arthritis was reported in a 15-day-old boy with wrist swelling who was successfully treated with 4 weeks of vancomycin [1].

The decision to administer a 6-day course of antibiotics in this case was based on the patient’s rapid clinical improvement following biliary drainage, normalization of inflammatory markers, and stable condition at discharge. However, in retrospect, this duration may have been inadequate for bacteremic biliary tract infection, particularly given the potential for endovascular seeding with *S. cristatus*. Ideally, the duration of therapy should be individualized, taking into account the causative organism, adequacy of source control, clinical response, and the exclusion of metastatic complications such as endocarditis.

The organism’s ability to cause invasive disease appears to be related to its capacity for biofilm formation and adherence to host tissues, although its specific virulence determinants remain poorly characterized. *S. cristatus* also influences oral microbial ecology and may exert indirect effects on pathogenicity under dysbiosis conditions [2].

Biliary tract infections are typically caused by enteric gram-negative bacteria, with *Escherichia coli* being the most common pathogen, followed by *Klebsiella* species [10], [11]. In cases of acute cholangitis, pathogenic microorganisms are commonly identified through blood cultures and bile cultures. A recent study reported that blood cultures were positive in approximately 40% of documented cases [12], with a higher detection rate in biliary stent obstruction, indicating increased sensitivity of blood cultures in this context [12]. Moreover, bile cultures showed a significantly higher positive rate, identified in 83% of cases [12].

Two primary routes have been proposed for bacterial contamination of bile: (1) ascending infection from the duodenum (duodenal microbiota), and (2) hematogenous spread via portal circulation [12]. In this case, the mechanism by which *S. cristatus* caused biliary tract infection was most likely hematogenous seeding from an oral source, similar to the pathogenesis described in reports of *S. cristatus* endocarditis [3]. Although the patient had no history of recent dental procedures or overt oral disease, diabetes mellitus may have served as a predisposing factor, as it is associated with increased susceptibility to infections and alterations in oral microbiota [6]. Additionally, choledocholithiasis with biliary obstruction provided a favorable environment for bacterial seeding and proliferation.

The antimicrobial susceptibility pattern observed in this case was consistent with previous reports on *S. cristatus* infections, which have generally demonstrated susceptibility to beta-lactam antibiotics. The effectiveness of penicillin, ceftriaxone, and vancomycin in treating such infections has been demonstrated in earlier studies [1], [3], [4], [5]. Similarly, the isolate in this study demonstrated favorable susceptibility. In our case, the patient received CMZ, a cephamycin antibiotic with activity against gram-positive and gram-negative bacteria, as well as anaerobes, similar to that of second-generation cephalosporins. In Japan, it is commonly used to treat urinary and biliary tract infections.

This case demonstrates significant clinical considerations, including diagnostic vigilance and the need to recognize emerging pathogens. Accurate species-level identification of VGS is important for epidemiological surveillance and appropriate antimicrobial selection. In this context, 16S rRNA gene sequencing improves the accuracy of identification compared with conventional biochemical methods. For VGS, which share similar biological characteristics, accurate identification using conventional methods is challenging. Moreover, the accuracy of identification by matrix-assisted laser desorption ionization–time of flight mass spectrometry (MALDI-TOF MS) varies depending on the reference library used [13], [14]. In this case, conventional methods were also unsuccessful.

This case report has limitations inherent to single-case studies. These constrains preclude establishing causality and prevent definitive guidance on optimal management strategies for this rare presentation. In this case, although gram-positive cocci were identified in blood cultures, treatment was guided by the assumption of infection with gram-negative rods, which are commonly found in the gastrointestinal tract. Consequently, the duration of antibiotic therapy may have been suboptimal, and neither echocardiography to exclude infective endocarditis nor repeat blood cultures were performed. The absence of echocardiography is a significant limitation, as subclinical valvular involvement cannot be definitively excluded. Similarly, the lack of repeat blood cultures precludes confirmation of bacteremia clearance. In addition, the absence of bile culture limits our ability to confirm *S. cristatus* as the primary causative agent of cholangitis, as opposed to secondary bacteremia from an oral source in the setting of biliary obstruction. Finally, delayed integration of microbiological results into clinical decision-making represents an important lesson from this case.

In summary, the present case represents the first documented instance of biliary tract infection with *S. cristatus* bacteremia. Accurate species-level identification is essential, as it provides insight into the pathogenic potential of organisms traditionally considered commensal.

## Abbreviations

AST, aspartate aminotransferase; ALT, alanine aminotransferase; ATCC, American Type Culture Collection; BLAST, Basic Local Alignment Search Tool; CMZ, cefmetazole; CRP, C-reactive protein; CT, computed tomography; ERCP, endoscopic retrograde cholangiopancreatography; EST, endoscopic sphincterotomy; H2, histamine-2; MALDI-TOF MS, matrix-assisted laser desorption ionization–time of flight mass spectrometry; MIC, minimum inhibitory concentration; rRNA, ribosomal ribonucleic acid; SAG, *Streptococcus anginosus* group; VGS, viridans group streptococci; WBC, white blood cell; γ-GTP, gamma-glutamyl transferase

## CRediT authorship contribution statement

**Ryosuke Nakamori:** Writing – review & editing, Methodology, Investigation. **Shohei Katsuno:** Writing – review & editing, Writing – original draft, Supervision, Project administration, Conceptualization. **Youta Takano:** Writing – review & editing, Methodology, Investigation.

## Consent for Publication

The patient provided written informed consent for publication.

## Ethics approval and consent to participate

This case report was conducted in accordance with institutional policies. Formal ethics committee approval was not required for this single case report, as per institutional guidelines. The patient provided written informed consent for the anonymous collection and use of his clinical data for research purposes.

## Funding

The authors received no specific funding for this work.

## Declaration of Competing Interest

The authors declare that they have no known competing financial interests or personal relationships that could have appeared to influence the work reported in this paper

## Data Availability

Not applicable.
